# BonA from *Acinetobacter baumannii* Forms a Divisome-Localized Decamer That Supports Outer Envelope Function

**DOI:** 10.1128/mBio.01480-21

**Published:** 2021-07-27

**Authors:** Rhys Grinter, Faye C. Morris, Rhys A. Dunstan, Pok Man Leung, Ashleigh Kropp, Matthew Belousoff, Sachith D. Gunasinghe, Nichollas E. Scott, Simone Beckham, Anton Y. Peleg, Chris Greening, Jian Li, Eva Heinz, Trevor Lithgow

**Affiliations:** a Infection and Immunity Program, Biomedicine Discovery Institute and Department of Microbiology, Monash University, Clayton, Australia; b Drug and Development Biology, Monash Institute of Pharmaceutical Sciences, Monash University, Parkville, Australia; c EMBL Australia Node in Single Molecule Science, University of New South Wales, Sydney, Australia; d Department of Microbiology and Immunology, University of Melbourne at the Peter Doherty Institute for Infection and Immunity, Melbourne, Australia; e La Trobe Rural Health School, College of Science, Health and Engineering, La Trobe University, Bendigo, Australia; f Department of Infectious Diseases, The Alfred Hospital and Central Clinical School, Monash University, Melbourne, Australia; g Liverpool School of Tropical Medicine, Liverpool, United Kingdom; Washington University in St. Louis; NIAID, NIH

**Keywords:** *Acinetobacter baumannii*, cell division, cell envelope, outer membrane proteins

## Abstract

Acinetobacter baumannii is a high-risk pathogen due to the rapid global spread of multidrug-resistant lineages. Its phylogenetic divergence from other ESKAPE pathogens means that determinants of its antimicrobial resistance can be difficult to extrapolate from other widely studied bacteria. A recent study showed that A. baumannii upregulates production of an outer membrane lipoprotein, which we designate BonA, in response to challenge with polymyxins. Here, we show that BonA has limited sequence similarity and distinct structural features compared to lipoproteins from other bacterial species. Analyses through X-ray crystallography, small-angle X-ray scattering, electron microscopy, and multiangle light scattering demonstrate that BonA has a dual BON (Bacterial OsmY and Nodulation) domain architecture and forms a decamer via an unusual oligomerization mechanism. This analysis also indicates this decamer is transient, suggesting dynamic oligomerization plays a role in BonA function. Antisera recognizing BonA shows it is an outer membrane protein localized to the divisome. Loss of BonA modulates the density of the outer membrane, consistent with a change in its structure or link to the peptidoglycan, and prevents motility in a clinical strain (ATCC 17978). Consistent with these findings, the dimensions of the BonA decamer are sufficient to permeate the peptidoglycan layer, with the potential to form a membrane-spanning complex during cell division.

## INTRODUCTION

Acinetobacter baumannii is a notorious “red alert” pathogen, considered an urgent threat to human health by international infectious disease control agencies (1–4). As a member of the gammaproteobacterial family *Moraxellaceae*, A. baumannii is genetically and physiologically divergent from well-studied model Gram-negative *Enterobacteriaceae* such as Escherichia coli. A. baumannii has a unique cell envelope that protects it from disinfectants and desiccation that readily kill other bacterial species, allowing it to persist for long periods on artificial surfaces in hospitals (5, 6). In addition, A. baumannii is notorious for its innate and acquired antibiotic resistance (2). It is currently estimated that as many as 50% of all A. baumannii infections in the United States are caused by strains resistant to carbapenems and many strains acquire polymyxin resistance during treatment (7, 8).

Like other Gram-negative bacteria, A. baumannii has a cell envelope consisting of an inner and outer membrane. This dual membrane encloses the periplasm, a crowded compartment that contains a thin layer of peptidoglycan (9). The outer membrane of A. baumannii is an intricate structure, consisting of an asymmetric lipid bilayer with an inner leaflet composed of phospholipids and an outer leaflet composed of lipooligosaccharide (LOS) (10). The LOS-derived surface of the outer membrane acts as a barrier to hydrophobic molecules (11). In addition to LOS and phospholipids, the outer membrane contains numerous proteins that are either integrated into or anchored in the membrane (12).

To maintain the integrity of the outer membrane, Gram-negative bacteria actively maintain its lipid asymmetry and coordinate its biogenesis rate with the overall rate of cell growth. In addition, the outer membrane must be constricted in conjunction with the peptidoglycan cell wall during division (13). To achieve this, Gram-negative bacteria have evolved a network of interlinked pathways for the construction and maintenance of the outer membrane (12, 14–22). Despite considerable progress in understanding how these pathways function in E. coli, in many cases, the proteins that constitute them are not well characterized, and additional pathways likely remain to be identified (12, 20, 23). In species divergent from E. coli, such as A. baumannii, these knowledge gaps are much more substantial.

Among these knowledge gaps is the role of dual-BON domain proteins, a widespread family of outer envelope proteins in Gram-negative bacteria. Dual-BON family proteins contain a pair of Bacterial OsmY and Nodulation (BON) domains, which fold into a conserved α/β sandwich (24). They possess a signal peptide targeting them to the periplasm, and some family members possess a lipobox with an N-terminal acylated cysteine, mediating peripheral outer membrane association (25, 26). They lack conserved residues indicative of an enzyme active site, though some family members bind phospholipids (27). Archetypical members of this dual-BON domain family are the outer membrane-associated lipid-binding protein DolP (formerly YraP) and the soluble periplasmic protein OsmY, both of which play a role in the construction and maintenance of the bacterial outer envelope (25–29). OsmY is an abundant periplasmic protein in E. coli induced in response to stressors such as osmotic shock, heat shock, acidic pH, and bile salts (25, 30). Recently, it was shown that OsmY functions as a chaperone, enhancing the stability of periplasmic proteins and the assembly of a subset of outer membrane proteins (31).

DolP is a lipoprotein widely present in Gram-negative bacteria. In E. coli and Neisseria meningitidis, it localizes to the inner leaflet of the outer membrane via an N-terminal lipid anchor (32–34). DolP was initially identified in E. coli as a lipoprotein whose expression is induced under cell envelope stress and it forms part of the σ^E^ regulon (35). Mutants of E. coli, N. meningitidis, and Salmonella enterica lacking DolP are compromised in outer membrane integrity, rendering the cells more sensitive to agents like the detergent sodium dodecyl sulfate (SDS) or the antibiotic vancomycin (26, 27, 29, 33, 35, 36). Possibly as a result of impaired outer membrane integrity, loss of DolP leads to attenuation of virulence in rodent models of infection (26).

There has been significant recent progress in determining the function of DolP in E. coli, suggesting multifaceted roles in regulating cell division and aiding outer membrane protein insertion during protein folding stress (27, 29, 32); however, the mechanism by which DolP mediates these functions remains to be elucidated. In E. coli, DolP is recruited to the site of cell division through interaction with anionic phospholipids, mediated α-helix 1 of its C-terminal BON domain (27, 32). This recruitment is required for the activation of cell wall remodeling enzymes during cell division, suggesting DolP has a regulatory role in this process (27, 32, 37). A recent study resolved the solution structure of DolP from E. coli, showing that it conforms to a dual-BON domain architecture and is monomeric (27). However, analysis of DolP isolated from E. coli membranes suggests that it forms oligomeric species (29). Furthermore, DolP interacts transiently with the BAM complex, which is responsible for the insertion of outer membrane proteins, and this interaction is important for coping with outer membrane protein folding stress (29). DolP association with the BAM complex is inversely correlated with localization to the site of cell division, suggesting a possible role for DolP in linking outer envelope stress to septal peptidoglycan hydrolysis (29).

The focus of this study is a dual-BON domain protein synthesized by A. baumannii. Previous work has shown that this bacterium can become resistant to the LOS-binding antibiotic polymyxin through mutations that prevent LOS production (38). These mutants survive with an outer membrane where phospholipids compose the only lipid species in both leaflets of the membrane (38). In both wild-type polymyxin-treated cells and polymyxin-resistant LOS-deficient mutants, a dual-BON domain family lipoprotein protein (HMPREF0010_02957 and ABBFA_002498) is upregulated (39, 40). This suggests that this protein, which we designate BonA, plays a role in adaption to the effects of polymyxin on the A. baumannii outer envelope and to the loss of LOS. BonA is only distantly related to either DolP or OsmY and we show that, unlike DolP, its loss in A. baumannii does not lead to a gross outer membrane permeability defect. However, A. baumannii mutants lacking BonA have an altered outer membrane structure and a defect in cell motility. Like DolP, single-cell imaging of A. baumannii indicates that BonA is localized to the divisome. However, BonA lacks conserved amino acids that mediate phospholipid binding by DolP (27), suggesting anionic phospholipids may not drive this localization. Through structural and biophysical analysis, we show that BonA forms a decamer and that this oligomerization is stabilized by a novel mechanism, involving rearrangement of the BON domain fold. Further, we show that BonA extracted from cell membranes is oligomeric and that the stability of the BonA oligomer is important for function in A. baumannii. Based on its unique structure, dynamic oligomerization, and role in optimal outer membrane function, we have identified BonA as an important component of the A. baumannii outer envelope.

## RESULTS

### BonA from *A. baumannii* is a member of a diverse family of dual-BON domain outer membrane lipoproteins.

**FIG 1:**
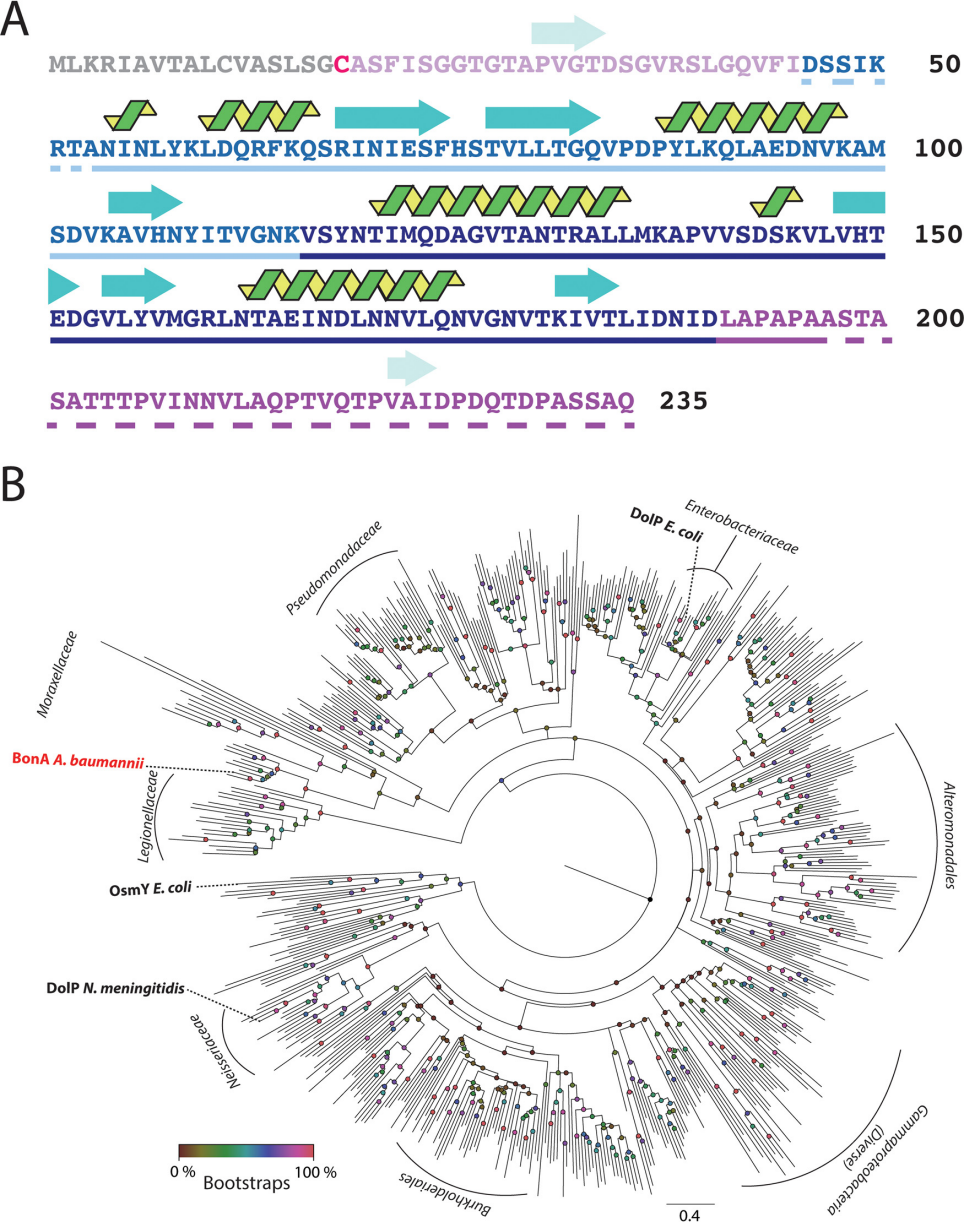
Sequence, secondary structure, and molecular phylogeny of BonA. (A) Amino acid sequence of BonA showing secondary structure (β-sheet = blue arrows, α-helix = green spirals; predicted or based on the BonA-27N crystal structure), the location of BON1 (light blue) and BON2 (dark blue), and regions largely lacking predicted structure (purple) and the signal peptide (gray) and acyl-anchored cysteine (red). Amino acids present in BonA-27N are underlined, solid for those resolved in the crystal structure, and dashed for disordered regions. (B) Maximum-likelihood phylogenetic tree of BonA homologs (shown in Table S1), showing the relatedness of BonA to the characterized family member DolP from E. coli and N. meningitidis. The clade containing the distinct dual-BON domain family member OsmY from E. coli was used to root the tree. Nodes are color-coded according to bootstrap values based on 100 replicates.

Analysis of A. baumannii genomes showed that they encode only one BON domain family protein, and sequence analysis of this lipoprotein suggests dual-BON domains, a terminal lipobox with an acyl-anchoring cysteine residue, and N- and C-terminal extensions (Fig. 1A). BonA shows substantial sequence divergence from DolP from E. coli (23% amino acid identity) and *Neisseria* spp. (24% identity) and is even more distantly diverged from OsmY from E. coli (20% identity) (see Table S1 in the supplemental material). A phylogenetic tree confirmed the distant evolutionary relationship between BonA and other dual-BON domain lipoproteins identified in a HMMER search of the reference proteome database (Fig. 1B; see also Table S1) (41). BonA belongs to a distinct clade clustering with proteins from other species of the family *Moraxellaceae*. A C-terminal proline-rich extension is present in BonA and other related sequences from Acinetobacter and *Moraxella* species but is absent from DolP and OsmY (Fig. 1A; see also Fig. S1).

10.1128/mBio.01480-21.1 FIG S1 Multiple sequence alignment of BonA from A. baumannii and DolP from E. coli and N. meningitidis. The proline-rich C-terminal extension present in BonA but absent from the E. coli and N. meningitidis proteins is notable. Serine residues resembling O-linked glycosylation sites in the BonA C-terminal extension are indicated with red arrows. Download FIG S1, JPG file, 0.4 MB. Copyright © 2021 Grinter et al. 2021 Grinter et al. https://creativecommons.org/licenses/by/4.0/ This content is distributed under the terms of the Creative Commons Attribution 4.0 International license.

10.1128/mBio.01480-21.6 TABLE S1 Sequence identity matrix of dual-BON family proteins from A. baumannii, E. coli, N. meningitidis and BonA homologs utilized for phylogenetic tree generation. Download Table S1, XLSX file, 0.1 MB. Copyright © 2021 Grinter et al. 2021 Grinter et al. https://creativecommons.org/licenses/by/4.0/ This content is distributed under the terms of the Creative Commons Attribution 4.0 International license.

### BonA is localized to the divisome and its deletion prevents motility.

**FIG 2:**
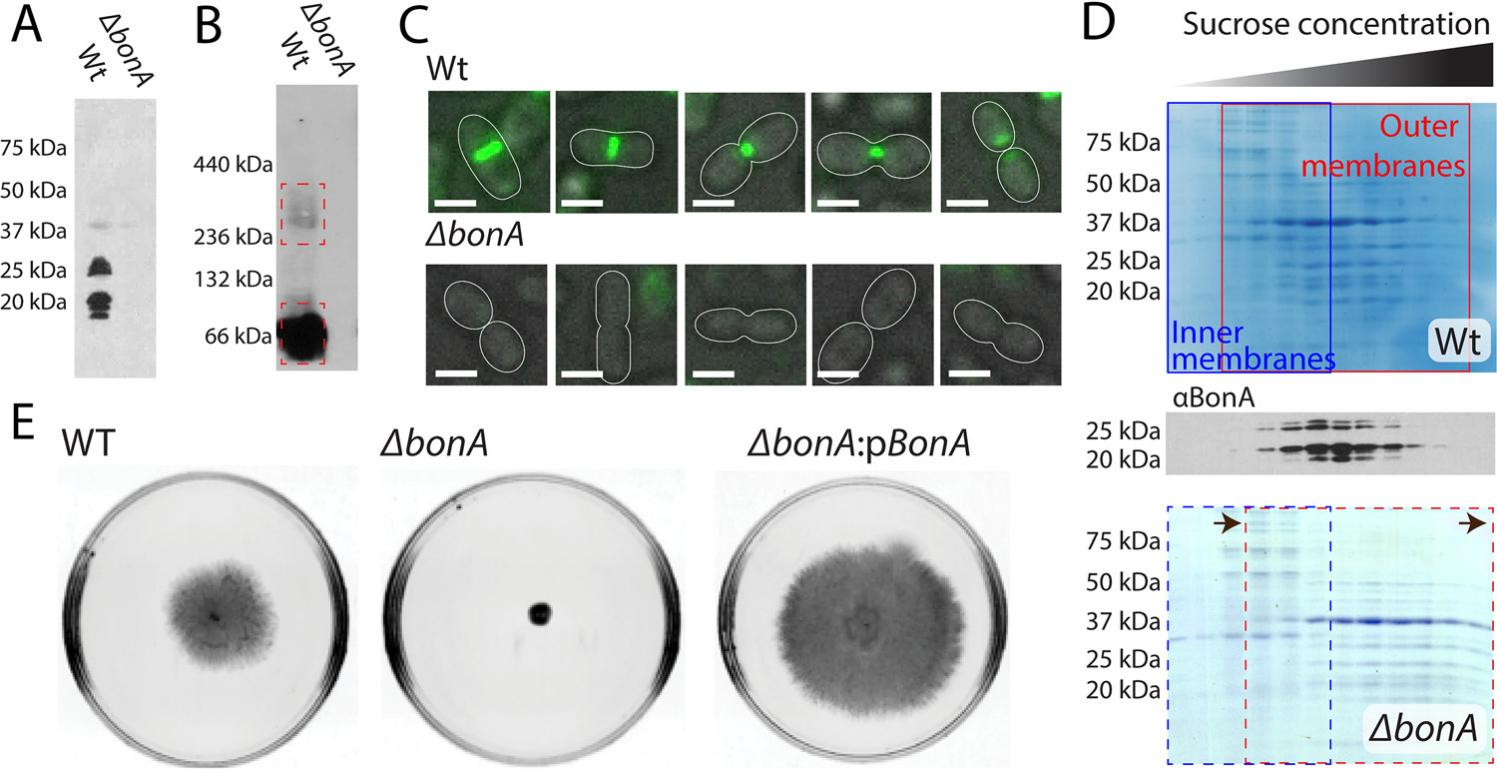
Cellular localization of BonA and phenotypes associated with loss of BonA in A. baumannii. (A) SDS-PAGE Western blot of total cellular membranes from wild-type and *ΔbonA* A. baumannii ATCC 19606 with an anti-BonA antibody, showing that BonA is membrane localized. (B) Blue-native PAGE Western blot of membranes as in panel A, showing BonA adopts a dimer and higher-molecular-weight species when purified from native membranes. (C) Immunofluorescence microscopy of wild-type and *ΔbonA* A. baumannii ATCC 19606 using an anti-BonA antibody, showing BonA is localized to the site of cell division; Scale bar, 2 μm. Cell outlines are traced to illustrate the cell division stage. (D) Coomassie blue staining and anti-BonA Western blot of SDS-PAGE of sucrose gradient separated of membranes from panels A and B showing that BonA is associated with fractions containing the outer membrane and that, in the *ΔbonA* strain, the outer membranes exhibit a higher density on the sucrose gradient. A difference between wild-type and *ΔbonA* mutant outer membrane densities was observed in four separate fractionation experiments, with the results of one representative experiment shown. (E) Semisolid motility assay plates of A. baumannii ATCC 17978, showing that the *ΔbonA* is nonmotile compared to the wild type and the complemented mutant, where expression of BonA from pWH1266 restores this phenotype.

The distant evolutionary relationship between BonA and other dual-BON family proteins poses the question of whether these proteins share a conserved function. To address this, we sought to determine the subcellular localization and physiological role of BonA. Mutants of the well-characterized A. baumannii type strain ATCC 19606 and clinical isolate ATCC 17978 were constructed (Δ*bonA*). Antibodies raised to BonA detected the protein in wild-type A. baumannii ATCC 19606, but not in the Δ*bonA* strain, when membrane extracts were analyzed by SDS-PAGE and immunoblotting (Fig. 2A). BonA was not detected in soluble cell extracts after ultracentrifugation, indicating it is entirely membrane-associated (see Fig. S2A). Upon Western blotting of BonA from membrane extracts, we noted multiple bands corresponding to the protein (Fig. 2A). This pattern is reminiscent of proteins modified by O-linked glycosylation mediated by the enzyme PglL, which glycosylates serine residues in motifs similar to those in the BonA C-terminal extension (see Fig. S1) (42, 43). To test whether O-linked glycosylation was present, we analyzed cell extracts from wild-type A. baumannii ATCC 17978 and a *pglL* deletion mutant (*ΔpglL*). In both strains the patterns of BonA on SDS-PAGE were analogous, suggesting that PglL does not glycosylate BonA (see Fig. S2B). The pattern of electrophoresis observed for BonA may be due to an unknown posttranslational modification or to proteolytic processing of the protein. To monitor the subcellular localization of BonA, cell membrane extracts were fractionated via a sucrose gradient, followed by immunoblotting, revealing that BonA colocalizes with the outer membrane porin Omp38 and not the inner membrane NADH-quinone oxidoreductase subunit NuoG (Fig. 2D; see also Fig. S2C).

10.1128/mBio.01480-21.2 FIG S2 Localization and phenotypic characterization of BonA containing A. baumannii. (A) Anti-BonA Western blot of fractionated A. baumannii ATCC 19606 cells showing BonA is associated with cellular membranes but not membrane-free ultracentrifuged supernatant. (B) Anti-BonA Western blot of fractionated wild-type and Δ*pglL* A. baumannii ATCC 17978 cells showing no difference in BonA banding, indicating that BonA is not glycosylated by PglL. (C) SDS-PAGE and Western blot of A. baumannii ATCC 19606 membranes separated by sucrose gradient, showing that BonA colocalizes with the outer membrane protein Omp38, not the inner membrane protein NuoG. Omp38 and NuoG were identified by MS-MS analysis. (D) Semisolid motility assay plates of A. baumannii ATCC 19606 showing that the wild-type strain is nonmotile and therefore that this phenotype is unaffected by the loss of BonA. Download FIG S2, JPG file, 0.8 MB. Copyright © 2021 Grinter et al. 2021 Grinter et al. https://creativecommons.org/licenses/by/4.0/ This content is distributed under the terms of the Creative Commons Attribution 4.0 International license.

While the relative abundance of proteins present in A. baumannii ATCC 19606 Δ*bonA* membranes was similar to the wild type, the outer membrane fraction from the Δ*bonA* strain progressed further into the sucrose gradient. This suggests that its structure or composition is altered, leading to an increase in density (Fig. 2D). No significant increase in sensitivity to SDS, vancomycin, or tetracycline was observed in the Δ*bonA* strain (see Table S2), suggesting that loss of BonA does not impair the integrity of the outer membrane in A. baumannii. A common phenotype for surface defects in A. baumannii is loss of motility on a swarm plate: while A. baumannii lacks flagella, twitching motility is observed in some strains of this species, thought to be mediated by the type IV pilus (44). Type IV pili are dynamic protein filaments that are assembled and secreted from the cell via a large protein complex that spans the bacterial cell envelope (45). ATCC 19606 is nonmotile, so this phenotype could not be tested in this strain (see Fig. S2D); however, loss of motility was observed in the A. baumannii ATCC 17978 *ΔbonA* strain, which could be complemented by the addition of *bonA* in *trans* (Fig. 2E). The loss of twitching motility observed in the ATCC 17978 *ΔbonA* mutant suggests that BonA plays either a direct or indirect role in the assembly or maintenance of the motility machinery.

10.1128/mBio.01480-21.7 TABLE S2 Susceptibility of *ΔbonA* A. baumannii strains to selected antimicrobial agents. Download Table S2, XLSX file, 0.01 MB. Copyright © 2021 Grinter et al. 2021 Grinter et al. https://creativecommons.org/licenses/by/4.0/ This content is distributed under the terms of the Creative Commons Attribution 4.0 International license.

Like BonA, DolP is a lipoprotein anchored to the outer membrane. DolP localizes to the divisome where it plays a role in regulating peptidoglycan remodeling during cell division (32). To determine whether BonA shares a common localization, we used the antibodies to monitor BonA in single cells via immunofluorescence microscopy. Consistent with localization to the divisome, fluorescence corresponding to BonA was observed as a central band in what appeared to be elongated, early-stage dividing cells. This band constricted in concert with the cell division septum (Fig. 2C; see also Fig. S3). No fluorescence beyond background was observed in *ΔbonA* cells (Fig. 2C; see also Fig. S3). To investigate the native structure of BonA, membrane extracts were solubilized in detergent and analyzed by blue-native PAGE. The vast majority of BonA was detected at a molecular weight of ∼60 kDa (Fig. 2B). Longer exposure of the immunoblots revealed a smaller proportion of BonA was detected as a larger oligomeric species (250 to 300 kDa) (Fig. 2B).

10.1128/mBio.01480-21.3 FIG S3 Immunofluorescence microscopy shows BonA localization to the midcell of dividing A. baumannii ATCC 19606. A representative wide-field image of wild-type and *ΔbonA* cells shows fluorescence associated with BonA, at the site of cell division in wild-type cells only. Dividing cells with BonA fluorescence are indicated by red boxes. Images are an overlay of phase contrast and fluorescence channels (green). Fluorescence observed in *ΔbonA* is a result of nonspecific antibody binding, since the cell line was confirmed to be non-BonA producing by Western blotting. Download FIG S3, JPG file, 2.2 MB. Copyright © 2021 Grinter et al. 2021 Grinter et al. https://creativecommons.org/licenses/by/4.0/ This content is distributed under the terms of the Creative Commons Attribution 4.0 International license.

### Crystal structure of BonA.

**FIG 3:**
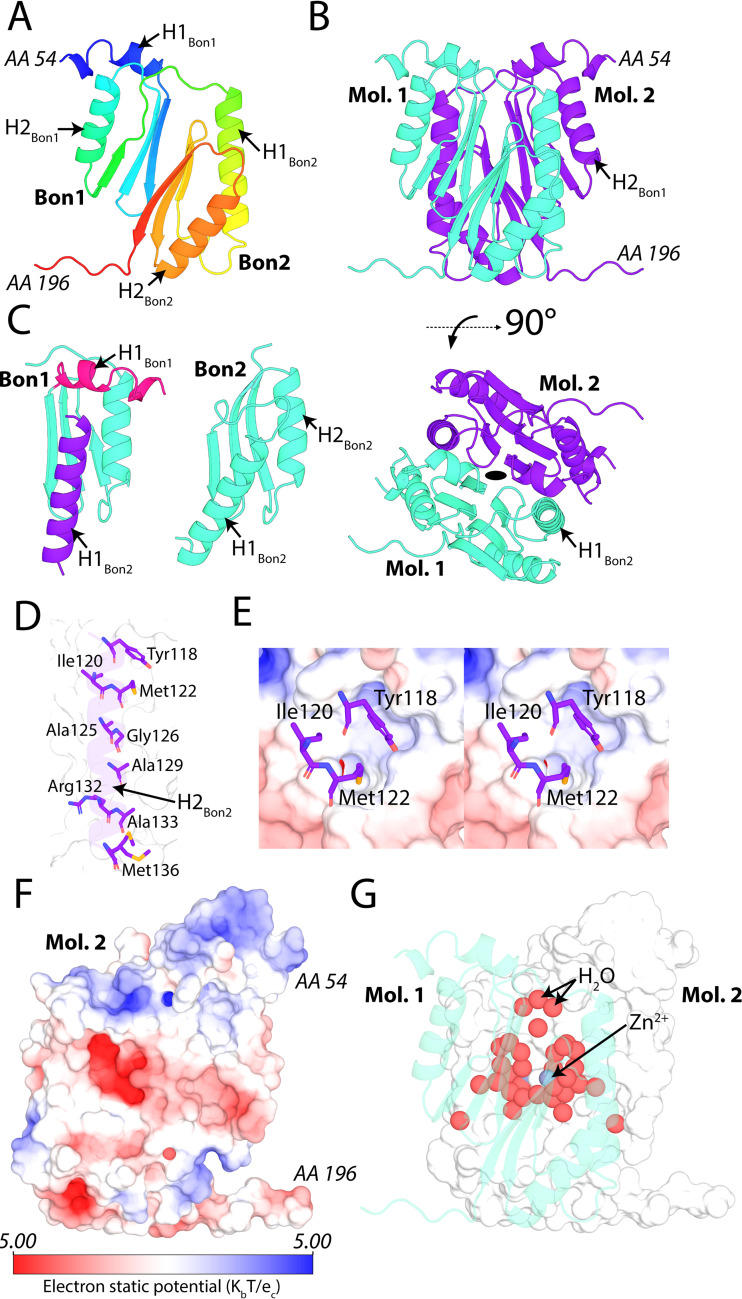
The crystal structure of BonA-27N reveals a dual-BON domain architecture that dimerizes via an α-helix swap mechanism. (A) The crystal structure of BonA-27N shown as a rainbow cartoon N terminus (blue) to C terminus (red) displays a dual-BON domain architecture with displaced α-helix 1 (αH1) of BON domain 1. (B) Dimer of BonA-27N observed *in crystallo*. (C) A key interface of the BonA-27N dimer involves the displacement of αH1 of BON1 by α-helix 1 (αH1) of BON2 of the opposing BonA molecule. αH1 of BON2 is amphipathic and interacts with the opposing molecule largely through hydrophobic interactions shown in panels D and E. As shown in panels F and G, the BonA dimer interface consists of both hydrophobic and polar interactions and is highly solvated.

To gain insight into the structural organization of BonA, as well as its architecture at the outer membrane, we solved its crystal structure. Crystal trials were performed with full-length BonA, as well as several truncation constructs. High-quality crystals were only obtained for N-terminally truncated BonA, missing the 27 amino acids (aa) after its lipid-anchoring cystine. The structure of this protein, designated BonA-27N, was solved at 1.65 Å by single-wavelength anomalous dispersion (SAD) phasing, using selenomethionine labeled protein. The structure of BonA-27N was built and refined from the resulting electron density maps (Fig. 3A; see also Table S3). The crystal structure of BonA-27N consists of two α/β-sandwich BON domains that interact extensively via the external face of their three-strand β-sheets (Fig. 3A). In contrast to the structure of DolP in which both domains adopt a canonical BON domain fold (27), in the BonA structure, α-helix 1 (αH1) of BON domain 1 (BON1) does not adopt the expected BON domain conformation of running parallel to the BON domain β-sheet. Rather, it is displaced from the rest of the domain (Fig. 3A). The 39 aa of the C-terminal extension of BonA-27N (aa 196 to 235) are disordered in the crystal structure. This region of BonA is not present in DolP or OsmY and is predicted to be largely unstructured (Fig. 1A; see also Fig. S1).

10.1128/mBio.01480-21.8 TABLE S3 Crystallographic and SAXS data collection and refinement statistics. Download Table S3, XLSX file, 0.01 MB. Copyright © 2021 Grinter et al. 2021 Grinter et al. https://creativecommons.org/licenses/by/4.0/ This content is distributed under the terms of the Creative Commons Attribution 4.0 International license.

Analysis of BonA-27N crystallographic symmetry reveals that it exists as a dimer, aligned with the crystallographic 2-fold axis (Fig. 3B). Analysis with the molecular interface prediction tool PISA (46) predicts that this interface is bona fide. The symmetrical BonA-27N dimer interacts via an extensive interface encompassing both BON domains (Fig. 3B). The interface is stabilized by αH1 of BON domain 2 (BON2), which substitutes for the displaced αH1 of BON1, thus completing the α/β-sandwich fold of BON1 (Fig. 3C). This interaction of αH1 of BON2 with BON1 is largely mediated by hydrophobic interactions (Fig. 3D), with Tyr118 and Met122 of αH1 of BON2 extending deeply into a hydrophobic pocket created by the displacement of αH1 of BON1 (Fig. 3E). While the interactions between αH1 of BON2 and BON1 are largely hydrophobic, the dimer interface of BonA-27N is mediated by a mixture of interaction types, including 14 hydrogen bonds and four salt bridges (Fig. 3F). The interface also contains two symmetrical, highly solvated pockets, which trap a total of 34 water molecules, as well as two Zn^2+^ ions which were present at a high concentration in the crystallization solution (Fig. 3G). In DolP from E. coli, αH1 of BON2 is responsible for binding to anionic phospholipids present in the outer membrane, with lipid binding mediating the divisome localization of DolP (27). In the BonA-27N structure, αH1 of BON2 is largely buried at the dimer interface and would be unable to access lipids. Further, BonA lacks conserved residues present in this helix of DolP required for lipid binding (27). This suggests that BonA localizes to the divisome by some other means.

A recent study by Wu et al. supports the physiological relevance of the BonA dimer, demonstrating via a global proteomic approach that interaction occurs between BonA molecules in A. baumannii cells (47). This study identified intermolecular cross-links between lysines 50, 59, and 65 of neighboring BonA molecules in A. baumannii cells. In the BonA-27N structure, lysines 59 and 65 are located in αH1 of BON1 and are within proximity to their dimer equivalent in our BonA-27N structure (see Fig. S4). Lysine 50 is unresolved in the crystal structure, but given this region of BonA is crucial for oligomerization, it is also a plausible candidate for cross-linking based on our data.

10.1128/mBio.01480-21.4 FIG S4 Lysine residues of BonA identified as interacting *in vivo* cross-linking conducted by Wu et. al. (47), shown on the structure of BonA-N27. Download FIG S4, JPG file, 0.2 MB. Copyright © 2021 Grinter et al. 2021 Grinter et al. https://creativecommons.org/licenses/by/4.0/ This content is distributed under the terms of the Creative Commons Attribution 4.0 International license.

### BonA decamerizes under physiological conditions through interactions mediated by its N-terminal extension.

**FIG 4:**
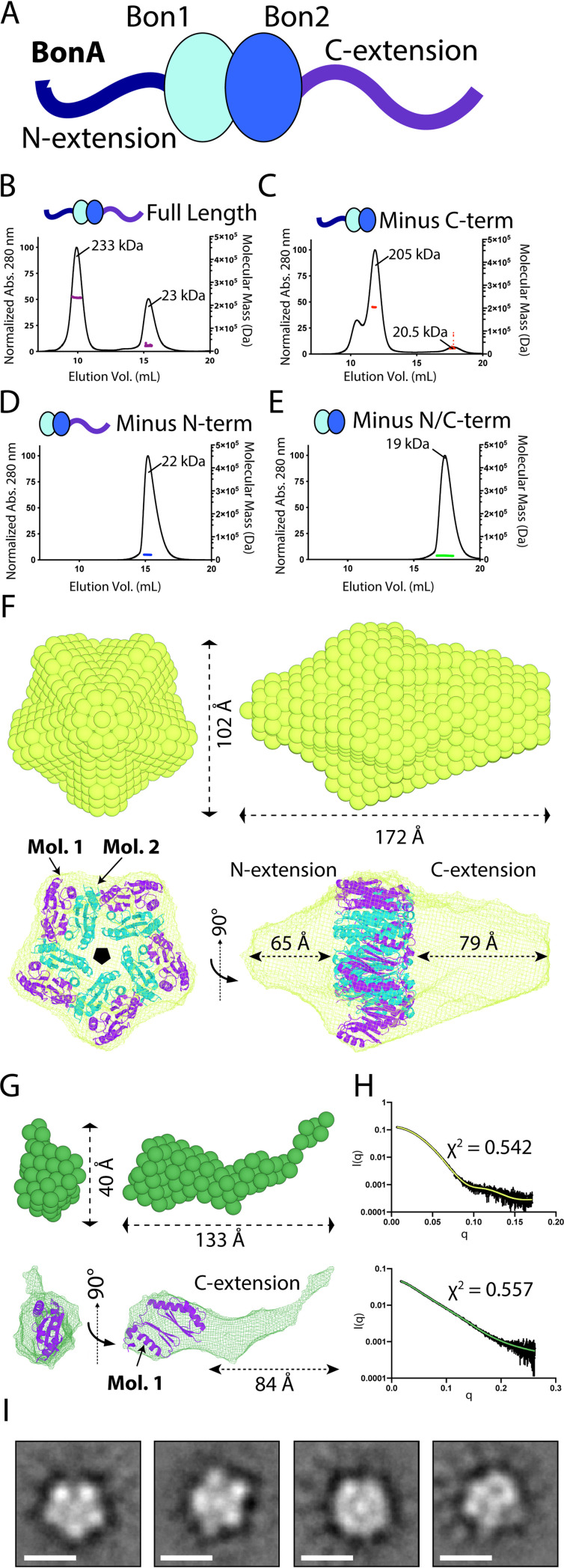
BonA forms a decamer with pentameric symmetry mediated by its N terminus. (A) A cartoon schematic of BonA showing its two central BON domains, with N- and C-terminal extensions with limited predicted secondary structure. (B and C) SEC-MALS experiments showing that full-length soluble BonA (B) and a 45-aa C-terminally truncated variant (C) are predominantly decamers, with some disassociation into a monomer. Conversely, 27-aa N-terminally truncated (D) and 45-aa C and 27-aa N-terminally truncated (E) variants both are monomers. (F) Bead model of the full-length BonA decamer modeled from SAXS data with C5 symmetry imposed (top), and a mesh representation of this bead model with the BonA-27N dimer structure modeled consistent with the observed decameric oligomerization (bottom). (G) Bead model of the BonA-27N monomer modeled from SAXS data (top), and a mesh representation of this bead model with monomer BonA-27N structure modeled. (H) SAXS scattering curves for full-length BonA (top) and BonA-27N (bottom) in black, with simulated scattering curves for the bead models in panels F and G shown in green. (I) Class averages generated from negative stain EM images of the cross-linked BonA decamer showing a pentameric organization; Scale bar, 100 Å.

Our structural and biochemical analysis indicated that BonA oligomerizes in A. baumannii cells and as a recombinant protein. To investigate the oligomeric state of BonA, the mature recombinant protein (lacking its signal sequence) was analyzed by size exclusion chromatography (SEC). In the absence of detergent, BonA migrated predominately as a high-molecular-weight species, with some disassociation to a lower-molecular-weight species observed. To gain a more precise understanding of the molecular weight of this oligomer, purified BonA was analyzed by analytical ultracentrifugation, revealing the presence of a single species with a molecular mass of ∼240-kDa (see Fig. S5A). The molecular mass of the BonA oligomer was confirmed by size exclusion chromatography coupled multiangle laser-light scattering (SEC-MALS), which indicated this species has a molecular mass of 233 kDa, which is consistent with a decamer, while the smaller species has a mass of 23 kDa, corresponding to a BonA monomer (Fig. 4B). To determine whether the N- or C-terminal extensions flanking the core BonA BON domains were responsible for oligomerization, truncation constructs lacking the N-terminal 27 aa succeeding the lipobox and/or the C-terminal 45 aa were analyzed via SEC-MALS (Fig. 1A and 4A). Removal of the C-terminal extension increased the tendency of BonA to aggregate but did not affect the oligomeric state of the protein, with a decamer of 205 kDa observed for the truncated protein (Fig. 4C). Conversely, loss of the 27 N-terminal amino acids abrogated oligomerization, with only a monomeric species of ∼22 kDa observed (Fig. 4D). Loss of both the N- and C-terminal regions also resulted in a monomeric protein, further confirming the role of the N terminus of BonA in oligomerization (Fig. 4E). In conclusion, BonA forms a decamer that requires its N-terminal extension and undergoes spontaneous disassociation into a monomeric species in solution. The monomeric nature of BonA-27N in solution contrasts with the dimer observed in its crystal structure, suggesting that weak interactions between monomers of this truncated protein are selected for during crystallization.

10.1128/mBio.01480-21.5 FIG S5 Full-length BonA forms a decameric oligomer consisting of discrete compact particles. (A) Analytical ultracentrifugation sedimentation profile for full-length BonA showing that it exists predominantly as a 240-kDa species, consistent with decameric oligomerization. (B) SDS-PAGE gel containing the eluted peak fraction from on column crosslinking of full-length BonA, with increasing concentration of the crosslinking reagent glutaraldehyde from left to right. (C) Representative negative-stain EM image of crosslinked full-length BonA from the highest glutaraldehyde concentration (0.5%) shown in panel B. (D to G) SAXS scattering curve for full-length BonA (D) and the Guinier (E), Kratky (F), and P(r) (G) plots derived from it. (H to K) SAXS scattering curve for BonA-27N (H) and the Guinier (I), Kratky (J), and P(r) (K) plots derived from it. Download FIG S5, JPG file, 0.8 MB. Copyright © 2021 Grinter et al. 2021 Grinter et al. https://creativecommons.org/licenses/by/4.0/ This content is distributed under the terms of the Creative Commons Attribution 4.0 International license.

To understand the basis of oligomerization of BonA, both full-length and BonA-27N were analyzed via size exclusion coupled small-angle X-ray scattering (SEC-SAXS) (see Fig. S5; see also Table S3). Despite the C-terminal extension, which largely lacks predicted secondary structure and was disordered in the BonA-27N crystal structure, SAXS scattering indicates that decameric BonA forms a compact particle in solution with maximum dimensions of ∼164 Å (see Fig. S5F and G). In contrast, SAXS scattering indicates that BonA-27N is highly flexible in solution with maximum dimensions of 107 Å, which is indicative of an unstructured and fully extended C terminus (see Fig. S5J and K). These differences between decameric and monomeric BonA suggest that intermolecular interactions stabilize the C terminus of the oligomeric form of the protein.

Molecular envelopes of full-length and BonA-27N were modeled based on SAXS scattering data. For full-length BonA, C5 symmetry was imposed, based on the decameric organization of the oligomer and the dimer observed in the crystal structure. The resulting molecular envelope was prolate, with dimensions of ∼172 by 102 Å. Five dimers of the BonA-27N crystal structure could be modeled with C5 symmetry into a bulge at the center of the envelope. The N and C termini of all molecules are orientated in the same direction, which is required by the lipid anchored N terminus of BonA. Regions truncated or disordered in the BonA-27N crystal structure could be accommodated by the remainder of the molecular envelope (Fig. 4F). The molecular envelope of BonA-27N was indicative of a monomer, with dimensions of ∼133 by 40 Å. The crystal structure of BonA-27N could be modeled into a bulge at one end of the envelope, with additional space accounting for the unstructured C-terminal extension (Fig. 4G). The simulated scattering curves for both envelopes were an excellent fit for the experimental data (Fig. 4H).

To validate the SAXS-based modeling of the BonA decamer, we further investigated BonA via negative-stain electron microscopy (NS-EM). Initial analysis of EM grids prepared with native BonA did not contain discrete particles. To stabilize the decamer, on-column glutaraldehyde cross-linking was performed, stabilizing BonA as first a dimeric and then a decameric species with increasing glutaraldehyde concentration (see Fig. S5B). NS-EM of the cross-linked sample revealed largely uniform monodisperse particles (see Fig. S5C). Two-dimensional (2D)-class averages derived from these images are suggestive of a particle with dimensions compatible with the BonA SAXS envelope and C5 symmetry, as predicted by other analyses (Fig. 4I).

### Disruption of the BonA dimer interface destabilizes the decamer and affects protein function.

**FIG 5:**
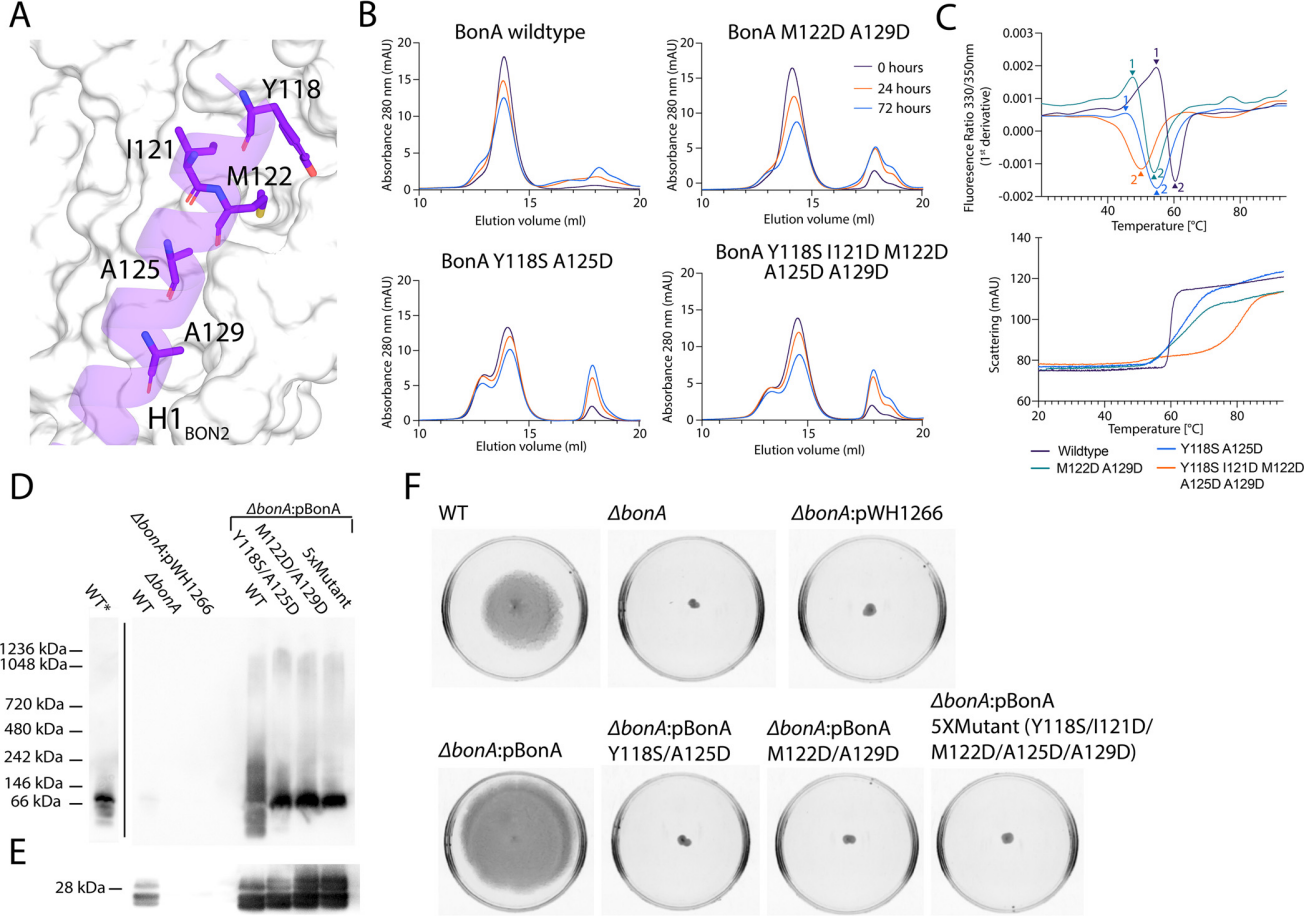
Dimer interactions mediated by BonA αH1 of BON2 are important for decamer stability and function in cells. (A) Amino acids of αH1 of BON2, shown as purple sticks, that form key hydrophobic interactions with the opposing molecule of the BonA dimer, shown as a white molecular surface. (B) SEC analysis of purified wild-type and mutant BonA at 0, 24, and 72 h postpurification, showing increased decamer dissociation of the mutants (monomer to decamer ratios shown in Table S4). (C) Differential scanning fluorimetry and light scattering of purified wild-type and mutant BonA as a function of temperature, showing first derivative transitions in the BonA 330/350-nm fluorescence ratio (top) and light scattering (bottom), indicating structural transitions of the protein. (D) Blue-native PAGE Western blot detecting BonA species in the membrane fraction of wild-type, *ΔbonA*, and wild-type and mutant BonA complemented strains. The lane designated WT* shows a longer exposure to visualize BonA in this sample. (E) SDS-PAGE Western blot detecting BonA in samples from panel D. (F) Semisolid motility assay plates of A. baumannii ATCC 17978, showing that complementation of the *ΔbonA* strain with of wild-type *bonA* restored the motility phenotype but complementation with the mutant variants does not.

To gain insight into the role of the BonA dimer in stabilizing the decamer, we expressed and purified a series of BonA variants with amino acid substitutions in hydrophobic residues of αH1 of BON2 that form part of the dimer interface (Fig. 5A). Two double substitutions (Y118S/A2125D and M122D/A129D) and one quintuple substitution (Y118S/I121D/M122D/A125D/A129D) were generated to maximize disruption of the BonA dimer interface. All mutant variants expressed and purified as the wild type, predominately decameric species were observed, with an increase in the amount of monomer present in all mutants. Wild-type and mutant BonA variants were purified concurrently and the decameric species were isolated and incubated for 0, 24, or 72 h at room temperature before the rate of decamer disassociation was monitored by SEC. All mutants displayed a higher rate of disassociation than the wild type, indicating that disruption of the BonA dimerization interface also partially destabilizes the BonA decamer (Fig. 5B; see also Table S4). The lack of total disruption of the decamer in the BonA mutants is consistent with the role of the BonA N-terminal extension in decamerization. The stability of the wild-type and BonA decamers was also tested using differential scanning fluorimetry and light scattering. Wild-type BonA fluorescence exhibited two inflection points at 54.3 and 60.5°C, the latter corresponding to an increase in light scattering indicating protein denaturation, with the former likely corresponding to decamer disassociation (Fig. 5C; see also Table S4). Inflection point 1 was shifted compared to wild type by −9 and −7°C for the Y118S/A2125D and M122D/A129D mutants, respectively, further indicating destabilization of the decamer in these mutants. Inflection point 1 was not observed for the quintuple mutant, and inflection point 2 was shifted by −10.4°C, indicating this mutant was further destabilized (Fig. 5C; see also Table S4).

10.1128/mBio.01480-21.9 TABLE S4 Decamer/monomer peak ratios and DSF inflection points for BonA wild-type and mutant proteins. Download Table S4, XLSX file, 0.01 MB. Copyright © 2021 Grinter et al. 2021 Grinter et al. https://creativecommons.org/licenses/by/4.0/ This content is distributed under the terms of the Creative Commons Attribution 4.0 International license.

To assess the effect of mutations on the function of BonA in A. baumannii, wild-type and mutant BonA constructs were introduced into the A. baumannii ATCC 17978 Δ*bonA* strain. The multiple copies of the complementation plasmid meant all complemented strains produced more BonA than wild-type A. baumannii ATCC 17978 (Fig. 5D and E). The migration pattern of the mutant BonA variants on blue-native PAGE was distinct from the wild-type complement strain, with a compact BonA species present at ∼66 kDa and less-diffuse species present at ∼240 kDa. However, the profile of the BonA mutants was more similar to that of the uncomplemented wild-type strain in this experiment, making the significance of these differences difficult to interpret. Interestingly, in all complemented strains a diffuse higher-order species at ∼1,000 kDa was observed, suggesting BonA is present in a larger oligomer or complex (Fig. 5D). As a readout for BonA functionality, the swarming motilities of wild-type, Δ*bonA*, and complemented strains were assessed (Fig. 5F). While motility was restored in A. baumannii ATCC 17978 Δ*bonA* complemented with wild-type BonA, none of the mutant BonA variants complemented the motility defect, indicating these variants have compromised functionality and that BonA oligomerization is important for function.

## DISCUSSION

**FIG 6:**
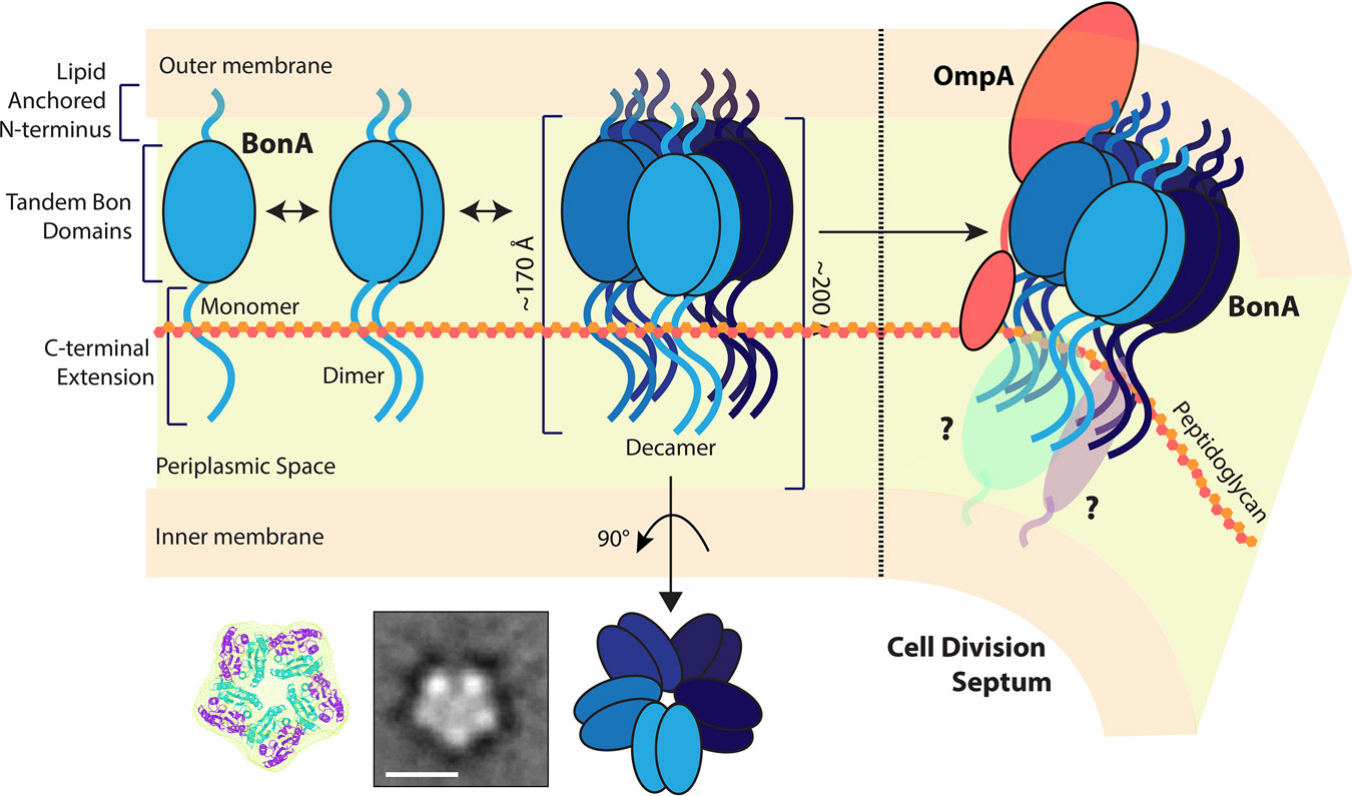
Model of BonA localization, oligomerization, and potential function at the outer membrane. BonA is anchored to the periplasmic side of the outer membrane, where it forms a transient decamer that spans the majority of the periplasmic space. BonA is recruited to the site of cell division, where it may interact with the peptidoglycan and act as a membrane-spanning scaffold for divisome proteins.

In this work, we identify BonA, a member of the bacterial dual-BON domain family of proteins, produced by A. baumannii and encoded by other members of the family *Moraxellaceae*. We demonstrate that BonA is anchored to the outer membrane where it plays a role in maintaining membrane structure and is required for twitching motility. Through structural analysis, we show that BonA possesses unique structural features and forms a divisome localized decamer that is important for the protein’s function (Fig. 6). We show that while BonA shares a common outer membrane and divisome localization to DolP from E. coli and *Neisseria* spp. (26, 28, 35), its loss does not lead to the gross defects in outer membrane permeability observed in DolP deletion mutants of these species. Further, purified DolP is monomeric and its divisome localization is mediated by phospholipid binding, while BonA is a decamer that lacks the conserved lipid-binding residues found in DolP (27). Analysis of DolP from E. coli membranes indicates it forms diffuse oligomers that echo those observed in membrane-derived BonA (29). However, whether the oligomerization mechanism we demonstrate for BonA extends to DolP and whether the conformation of monomeric BonA is analogous to DolP remain to be determined. Furthermore, whether, like DolP, BonA interacts transiently with the BAM complex to mitigate protein folding stress is an interesting question for future study (29).

The change in outer membrane density associated with the loss of BonA suggests a significant alteration in the structure or composition of this membrane or the physical membrane-peptidoglycan links. Consistent with this, the loss of twitching motility observed in the A. baumannii ATCC 17978 *ΔbonA* mutant, likely mediated by an outer-envelope spanning type IV pilus (44), is suggestive of a perturbed outer envelope. These data are also consistent with our previous finding that BonA is upregulated in response to outer membrane destabilizing polymyxins (38, 39) and broadly indicate a role for BonA in supporting optimal outer membrane function.

While further work is required to determine the precise role of BonA in the outer membrane, our structural analysis provides important insights into BonA function. We show that BonA forms a decamer that is ∼172 Å in length. In the context of the periplasmic space, where the nominal distance between the outer and inner membranes is ∼200 Å (48), outer membrane-anchored BonA would span the majority of the periplasm if extending perpendicular from the membrane (Fig. 6). In this configuration, BonA would penetrate the peptidoglycan layer and would be capable of interacting with proteins embedded in the inner membrane, thus bridging the inner and outer membranes. When localized to the site of cell division, BonA could tether the outer membrane to the peptidoglycan or the membrane-spanning divisome complex (Fig. 6). In support of this hypothesis, in-cell cross-linking data show interactions occur between BonA and OmpA in A. baumannii (47), with OmpA playing a role in tethering the outer membrane to the peptidoglycan (49). The transient nature of BonA oligomerization is also consistent with a role in coordinating a dynamic process during cell division. If BonA is indeed important for coordinating the outer envelope during cell division, its loss would lead to improper remodeling of this structure, which is consistent with the *ΔbonA* phenotypes we observe.

The cell envelope provides a key defense for A. baumannii against antimicrobial compounds and environmental stress. To effectively combat A. baumannii infection and its persistence in the hospital environment, we must develop strategies to overcome the outer envelope’s defenses. To do so, a robust understanding of the key factors required for outer membrane construction and maintenance is required. Our work on BonA informs this understanding and provides insights into the role of this protein in supporting outer membrane function in A. baumannii.

## MATERIALS AND METHODS

### Protein sequence analysis.

To determine the relationship between distantly related dual-BON domain family members, we constructed a tree of BonA homologs, identified with a HMMER search of the reference proteomes database using BonA as a query sequence (41). BonA homologs identified in the HMMER search were curated to only include proteins with a dual-BON domain architecture and a lipobox sequence determined using SignalP 5.0 (50). This yielded 896 sequences, which were further reduced for tree construction using CD-Hit to filter sequence with a pairwise similarity of <75%, yielding 565 sequences (see Table S1) (51). These 565 protein sequences, plus OsmY from E. coli as a sequence to define the root branch, were aligned using MUSCLE (52) implemented in the phylogenetic analysis program MEGAX (v.10.1.7) (53), which was subsequently used as the input for constructing a maximum-likelihood (ML) phylogenetic tree to infer evolutionary relationships for this protein family. The best amino acid substitution model was inferred using MEGAX which compared 56 different models; the Jones-Taylor-Thornton model with a gamma distribution of five discrete gamma categories and invariant sites (G+I) was selected. To infer tree topology, the default ML heuristic method ML nearest-neighbor-interchange was applied, and initial trees were made with Neighbor-Joining and BioNJ algorithms. The final tree was built by including all residues and bootstrapping with 100 replicates.

### Strain propagation, maintenance, and antimicrobial susceptibility testing.

E. coli and A. baumannii strains were propagated in lysogeny broth (LB) and LB agar at 37°C, with antibiotic selection to maintain plasmids where appropriate. Antimicrobial susceptibility was conducted per CLSI guidelines using the broth microdilution method and cation adjusted Muller Hinton broth. MICs were defined as >80% reduction in growth, and significance considered as >2 concentration increase or decrease in MIC relative to the wild-type control.

### Construction of *ΔbonA* strains in *A. baumannii*.

Plasmid DNA, genomic DNA, and PCR products were purified using relevant kits from Bioneer, Qiagen, and Promega, respectively, according to the manufacturers’ instructions. The A. baumannii *ΔbonA* mutants were constructed as described previously (54), with minor modifications. Briefly, the kanamycin resistance cassette was PCR amplified from pKD4 using disruption primers containing >80 bases of homology to the *bonA* flanking sequence (as described in Table S5 in the supplemental material). The resultant fragments were gel purified and introduced into A. baumannii strains ATCC 17978 and ATCC 19606 by electroporation as previously described (55), with selection on LB agar, supplemented with 50 μg/ml kanamycin. The mutations were confirmed by PCR amplification using primers flanking the insertion, followed by Southern hybridization of genomic DNA digested with EcoRV, probed with kanamycin- and *bonA*-specific digoxigenin-labeled probes, as described previously (56).

10.1128/mBio.01480-21.10 TABLE S5 Primers and strains utilized for this study. Download Table S5, XLSX file, 0.01 MB. Copyright © 2021 Grinter et al. 2021 Grinter et al. https://creativecommons.org/licenses/by/4.0/ This content is distributed under the terms of the Creative Commons Attribution 4.0 International license.

For complementation, the full-length *bonA* sequence plus 500 nucleotides upstream of the translational start site (deemed to include the native promoter) were PCR amplified from ATCC 17978 with forward and reverse complementation primers encoding 5′ AatII and EcoRI restriction sites, respectively. The resultant fragments were digested and ligated into the E. coli*-*Acinetobacter shuttle vector, pWH1266 (57). To create Y118S/A125D, M122D/A129D, and Y118S/I121D/M122D/A125D/A129D mutant BonA complementation constructs, gene blocks identical to the PCR-amplified sequence, aside from the stated mutations, were synthesized and cloned into pWH1266. The p*BonA* wild-type and mutant constructs were confirmed by sequencing before electroporation into the respective mutant strains as described previously, with the pWH1266 vector-only used as a control.

### Twitching motility assays.

Twitching motility was assessed as described previously (44). Briefly, a 1-μl drop of stationary-phase culture was placed onto the center of a 0.25% modified LB agarose and incubated at 37°C for up to 48 h. Three independent experiments were performed for each.

### BonA antiserum generation.

Polyclonal rabbit antiserum for the detection of BonA was generated at the Monash Animal Research Platform from recombinant proteins purified in-house. Rabbits were serially injected with purified protein (10 mg/ml) in combination with complete (first injection) or incomplete (subsequent injections) Freund adjuvant, over 1 to 3 months, with clarified rabbit sera periodically tested for reactivity to the target protein. Once acceptable levels of reactivity were achieved, rabbits were euthanized, and clarified sera were collected and stored at −80°C.

### Isolation and fractionation of membranes from *A. baumannii*.

A. baumannii cells were cultured in LB media and grown to an optical density at 600 nm (OD_600_) of ∼0.6 before harvesting. Membranes were purified and subsequently fractionated by sucrose density fractionation (60:55:50:45:40:35% [wt/wt]) as described previously (58).

### Detection and localization of BonA in *A. baumannii* cell extracts by Western blotting.

For the detection of BonA in cell extracts, 50-μg portions of isolated total membranes were analyzed by 10% SDS-PAGE or 5 to 16% blue-native (BN)-PAGE (59) and was subsequently analyzed by Western blotting against BonA (antibody dilution, 1:20,000). To determine the cellular localization of BonA, 30-μl aliquots of each fraction from the sucrose gradient were separated by 10% SDS-PAGE for Coomassie blue staining and subsequent Western blotting as described above.

### Localization of BonA in *A. baumannii* cells by immunofluorescence microscopy.

Bacterial cultures were grown to mid-log phase in LB media at 37°C with shaking (200 rpm). Then, 500 μl of culture medium was centrifuged (4,000 × *g*, 5 min, 4°C), washed twice in phosphate-buffered saline (PBS), and resuspended in 500 μl of PBS. Eight-well, coverglass-bottom chambers (Sarstedt) were coated with 0.01% (vol/vol) poly-l-lysine (Sigma-Aldrich, P8920) for 10 min at room temperature before excess poly-l-lysine was removed. Afterward, 200 μl of bacterial cell suspension was immobilized onto each well. To ensure a monolayer of bacteria was formed at the bottom of each well, chamber slides were subjected to centrifugation (4,000 × *g*, 3 min, 4°C), followed by several washing steps to remove nonadhered cells. The monolayer of bacteria was then fixed with a mixture of paraformaldehyde (2% [wt/vol]) and glutaraldehyde (0.2% [vol/vol]) in PBS for 5 min at 4°C, which was then washed with PBS to remove excess fixatives. To reduce autofluorescence caused by the background, samples were treated with the fluorescence quencher NaBH_4_ at a concentration of 0.1% (wt/vol) in PBS, followed by several washing steps with PBS. Samples were then permeabilized with Triton X-100 (0.001% [vol/vol] in PBS), followed by three washing steps with PBS.

Before antibody staining, samples were blocked with 5% (wt/vol) bovine serum albumin (BSA) in PBS for 1 h at room temperature, followed by incubation with anti-BonA antisera (1:1,000 in 5% [wt/vol] BSA in PBS) for 1-h mixing by rotary inversion at room temperature. Samples were washed thoroughly with PBS to remove excess antiserum. Secondary staining was carried out for 45 min at room temperature using anti-rabbit immunoglobulin G (IgG)-Alexa Fluor 488 (Thermo Fisher, A-11008) diluted to 1:3,000 (in 5% BSA in PBS), followed by several washing steps to remove excess antibody. Olympus IX-81 inverted fluorescence microscope equipped with Olympus Cell˄M software was used to visualize bacterial samples using a 100× objective with a fluorescein isothiocyanate filter.

### Protein expression and purification.

DNA encoding full-length BonA and Bon*A*-C45 were amplified by PCR, with C-terminal NcoI and XhoI restriction sites and cloned into a pET20b derived vector which added a 10× N-terminal His tag, followed by a TEV cleavage site, via restriction digest and ligation. Gene blocks encoding Y118S/A125D, M122D/A129D, and Y118S/I121D/M122D/A125D/A129D mutant full-length *bonA* constructs were synthesized and cloned into the modified pET20b derived vector as for the wild-type protein. The resulting vector was transformed into E. coli BL21(DE3) C41 cells. DNA encoding BonA-27N and BonA-27N-45C were amplified by PCR, minus stop codon, with C-terminal NdeI and XhoI restriction sites and cloned into pET22b vector which added a 6× C-terminal His tag. The resulting vectors were transformed into E. coli BL21(DE3) C41 cells. Protein expression was performed in terrific broth (12 g tryptone, 24 g yeast extract, 61.3 g K_2_HPO_4_, 11.55 g KH_2_PO_4_, 10 g glycerol) with 100 mg/ml ampicillin for selection. Cells were grown at 37°C until they reached an OD_600_ of 1.0 induced using 0.3 mM IPTG (isopropyl-β-d-thiogalactopyranoside), followed by further growth 14 h at 25°C. For selenomethionine-labeled BonA-27N, the BonA-27N construct was transformed into the methionine auxotrophic E. coli strain Crystal Express (DE3). Cells were grown in M9 minimal medium containing 100 mg/liter of each amino acids (minus methionine) and 50 mg/liter selenomethionine. Cells were harvested by centrifugation, lysed using a cell disruptor (Emulsiflex) in Ni-binding buffer (50 mM Tris, 500 mM NaCl, 20 mM imidazole [pH 7.9]) plus 0.1 mg/ml lysozyme, 0.05 mg/ml DNase I, and cOmplete protease cocktail inhibitor tablets (Roche). The resulting lysate was clarified by centrifugation and applied to Ni-agarose resin, followed by washing with 10× column volumes of Ni-binding buffer, and elution of bound proteins with a step gradient of Ni-gradient buffer (50 mM Tris, 500 mM NaCl, 500 mM imidazole [pH 7.9]) of 5, 10, 25, and 50%. Eluted fractions containing recombinant protein were pooled and applied to a 26/600 Superdex S200 size exclusion column equilibrated in SEC buffer (50 mM Tris, 200 mM NaCl [pH 7.9]). The recombinant protein was then pooled concentrated to 10 mg/ml, snap-frozen, and stored at −80°C.

### Size-exclusion chromatography multiangle light scattering.

The absolute molecular masses of BonA-FL and truncated variants were determined by SEC-MALS. First, 100-μl protein samples (1 to 5 mg/ml) were injected onto a Superdex 200 10/300 GL size exclusion chromatography column in 20 mM Tris–200 mM NaCl (pH 7.9) at 0.6 ml/min with a Shimadzu LC-20A. The column eluent was fed into a DAWN Heleos II MALS detector (Wyatt Technology), followed by an Optilab T-rEX differential refractometer (Wyatt Technology). Light scattering and differential refractive index data were collected and analyzed using ASTRA 6 software (Wyatt Technology). Molecular masses and estimated errors were calculated across individual eluted peaks by extrapolation from Zimm plots with a refractive index increment (dn/dc) value of 0.1850 ml/g. SEC-MALS data are presented with the absorbance (280 nm) plotted alongside fitted the molecular masses (*M*_r_).

### Analytical size exclusion chromatography of wild-type mutant BonA.

Wild-type and Y118S/A125D, M122D/A129D, and Y118S/I121D/M122D/A125D/A129D mutant BonA was purified concurrently as described above. Protein concentration was normalized to 3 mg/ml, and 200 μl was loaded onto a Superdex S200 Increase 10/300 column equilibrated in 50 mM Tris–200 mM NaCl (pH 7.9) after 0, 24, and 72 h of incubation at 22°C. Chromatograms were analyzed based on known decamer and monomer peaks, and the peak areas were calculated.

### Differential scanning fluorimetry of wild-type mutant BonA.

Fluorescence at 330 and 350 nm and light scattering of wild-type and mutant BonA were recorded as a function of temperature using a Prometheus NanoDSF instrument, with a BonA concentration of 5 mg/ml in 50 mM Tris–200 mM NaCl (pH 7.9). Due to the lack of tryptophan residues in BonA, the fluorescence recorded resulted from tyrosine residues.

### Protein crystallization, data collection, and structure solution.

Purified BonA proteins were screened for crystallization conditions using commercially available screens (∼800 conditions). Crystals grew from drops containing BonA-27N in medium composed of 0.2 M zinc acetate, 0.1 M sodium acetate, and 20% PEG 3350 (pH 4.5), and the crystals were optimized from this starting condition. The crystals were cryoprotected by increasing PEG 3350 concentration to 30% and flash cooled in liquid N_2_. Diffraction data were collected at 100 K at the Australian Synchrotron on selenomethionine-labeled crystals and processed in the space group P3_1_21 to 1.65 Å. Heavy atom sites were located, phases were obtained using single-wavelength anomalous dispersion (SAD), and the initial model was built using Autosol from the Phenix package (60). Eight heavy atom sites were located; four of these sites were selenium, and four of these sites were zinc. The BonA-N27 model was improved manually in Coot and refined using Phenix refine and Refmac (60–62). Analysis of the BonA-27N crystal structure was performed using the Phenix and CCP4 packages, noncrystallographic interfaces were predicted using PISA (46, 60, 63).

### Small-angle X-ray scattering.

Small-angle X-ray scattering (SAXS) was performed using Coflow SEC-SAXS at the Australian Synchrotron (64). Purified BonA and BonA-27N were analyzed at a preinjection concentration of 10 mg/ml. Scattering was collected over a *q* range of 0.0 to 0.3 Å^−1^. A buffer blank for each SEC-SAXS run was prepared by averaging 10 to 20 frames before or after protein elution. Scattering data from peaks corresponding to BonA and BonA-27N were then buffer subtracted and scaled across the elution peak and compared for interparticle effects. Identical curves (5–10) from elution were then averaged to provide curves for analysis. Data were analyzed using the PRIMUS package, ScÅtter, and DAMMIF modeler (65).

### Analytical ultracentrifugation.

Sedimentation velocity (SV) determination was carried out in a Beckman Coulter Optima analytical ultracentrifuge using an An-50 Ti 8-hole rotor. BonA-FL (370 μl) at concentrations ranging from 0.25 to 2 mg/ml was loaded into a 12-mm path-length centerpiece and centrifuged at 40,000 rpm for ∼6 h at 20°C. Scans were collected every 20 s using absorbance optics (at 230, 240, and 280 nm, with a radial range of 5.8 to 7.2 cm and a radial step size of 0.005 cm). We used 50 mM Tris–200 mM NaCl (pH 7.9) as the buffer. Data were analyzed with SEDFIT using the continuous c(s) distribution model (66). SEDNTERP was used to calculate the partial specific volume, the buffer density, and the viscosity at 15 and 20°C.

### On-column cross-linking and negative-stain electron microscopy.

To stabilize the BonA decamer an “on-column” cross-linking method was used. Initially, 200 μl of glutaraldehyde solution (0.05 to 0.5% in dH_2_O) was injected to a preequilibrated Superdex 200 10/300 column in buffer (20 mM HEPES, 150 mM NaCl [pH 7.4]). The column was run at 0.25 ml /min for 20 min (5 ml buffer). Subsequently, the column flow was paused, and the injection loop was flushed using buffer, followed by injection of purified BonA (200 μl at 10 mg/ml). Subsequently, the column was run at 0.25 ml/min, and 0.5-ml fractions were collected. Collected fractions were immediately quenched by the addition of 50 μl of 50 mM Tris (pH 7.5). The crosslinking efficiency was visualized by running the individual fractions on a 10% SDS gel, and cross-linked fractions were flash-frozen for NS-EM analysis.

Native and cross-linked BonA were serially diluted in buffer (20 mM HEPES, 150 mM NaCl [pH 7.4]), and 5 μl was spotted onto freshly glow-discharged carbon-coated 200-mesh copper grids (Pelco), followed by blotting to remove all but a thin film of protein solution. Blotted grids were fixed with the tungsten-based Nano-W strain (Nanoprobes) by adding the stain to each grid, followed by 60 s of incubation and blotting; this was repeated three times prior to air drying. The grids were imaged on a 120-keV Tecnai Spirit G2 microscope (FEI) equipped with a 4 K FEI Eagle camera. Images were processed, particles were picked, and 2D classes were generated using the RELION package (v2.1) (67).

### Data availability.

The crystallographic coordinates and associated structure factors for BonA are available at the Protein Data Bank (PDB) under accession code 6V4V. Small-angle X-ray scattering data for BonA full-length and BonA-27N are available in the SASBDB with the accession codes SASDJW3 and SASDJX3. The accession numbers of protein sequences used to construct the phylogenetic tree are provided in Table S1 in the supplemental material.
