# Supplementary figures and images for: BonA from Acinetobacter baumannii Forms a Divisome-Localized Decamer That Supports Outer Envelope Function
Source: mBio. 2021 Jul 27;12(4):e01480-21. doi: 10.1128/mBio.01480-21 (PMC8406262; doi:10.1128/mBio.01480-21)

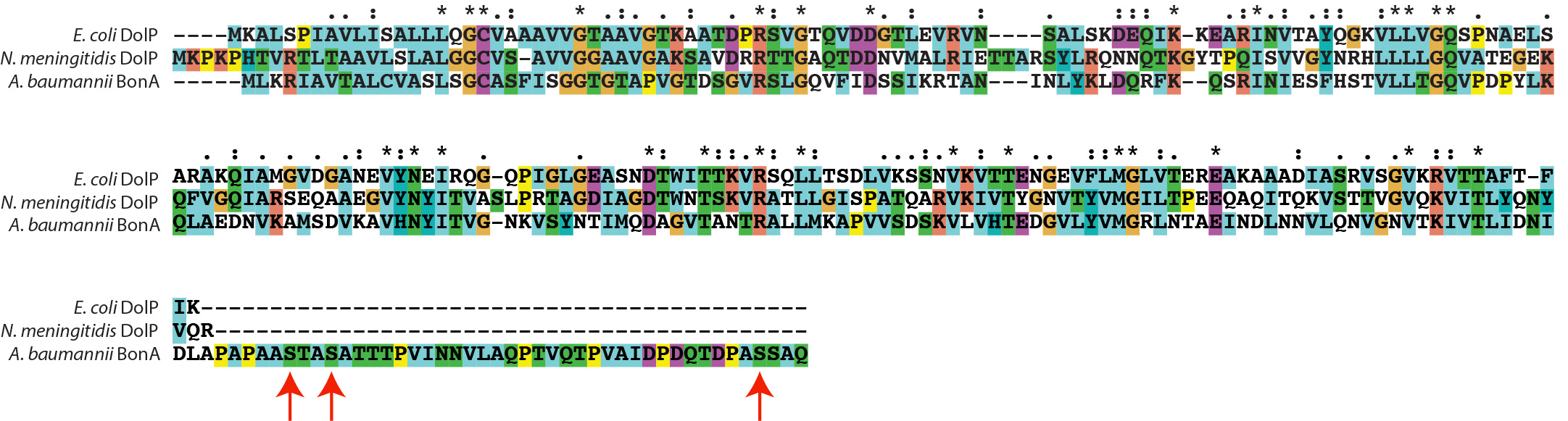

Supplement: FIG S1 [file mbio.01480-21-sf001.jpg]

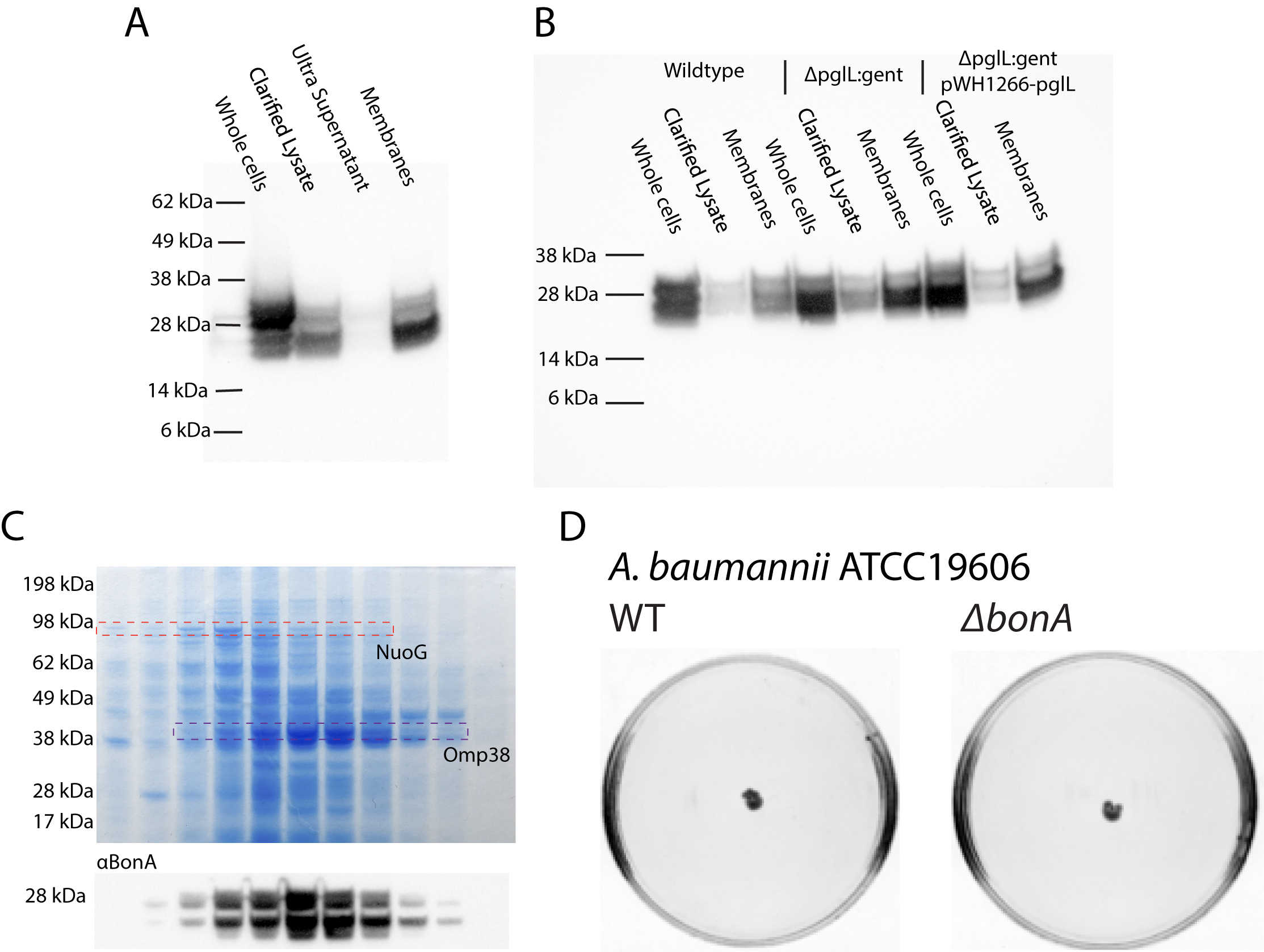

Supplement: FIG S2 [file mbio.01480-21-sf002.jpg]

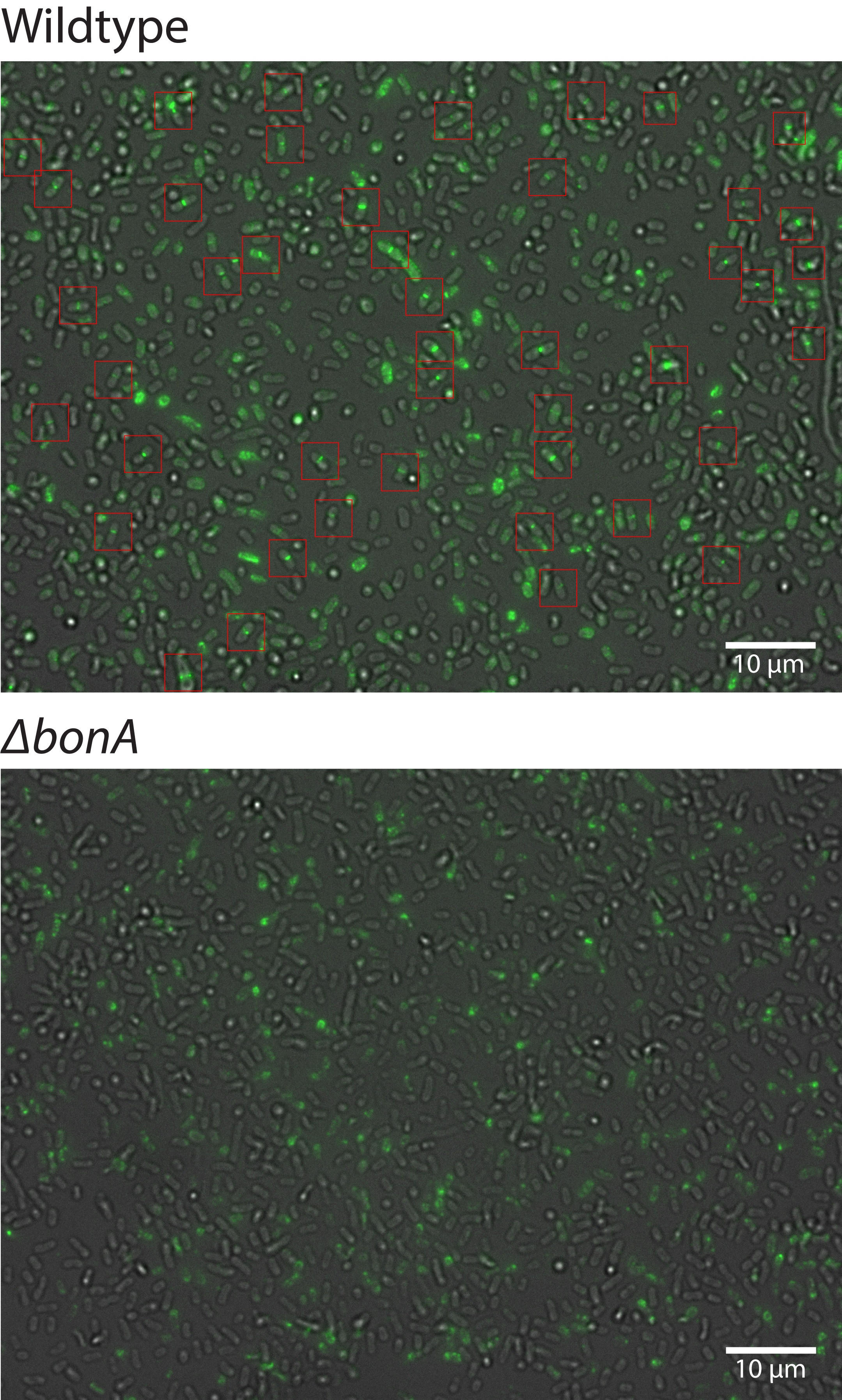

Supplement: FIG S3 [file mbio.01480-21-sf003.jpg]

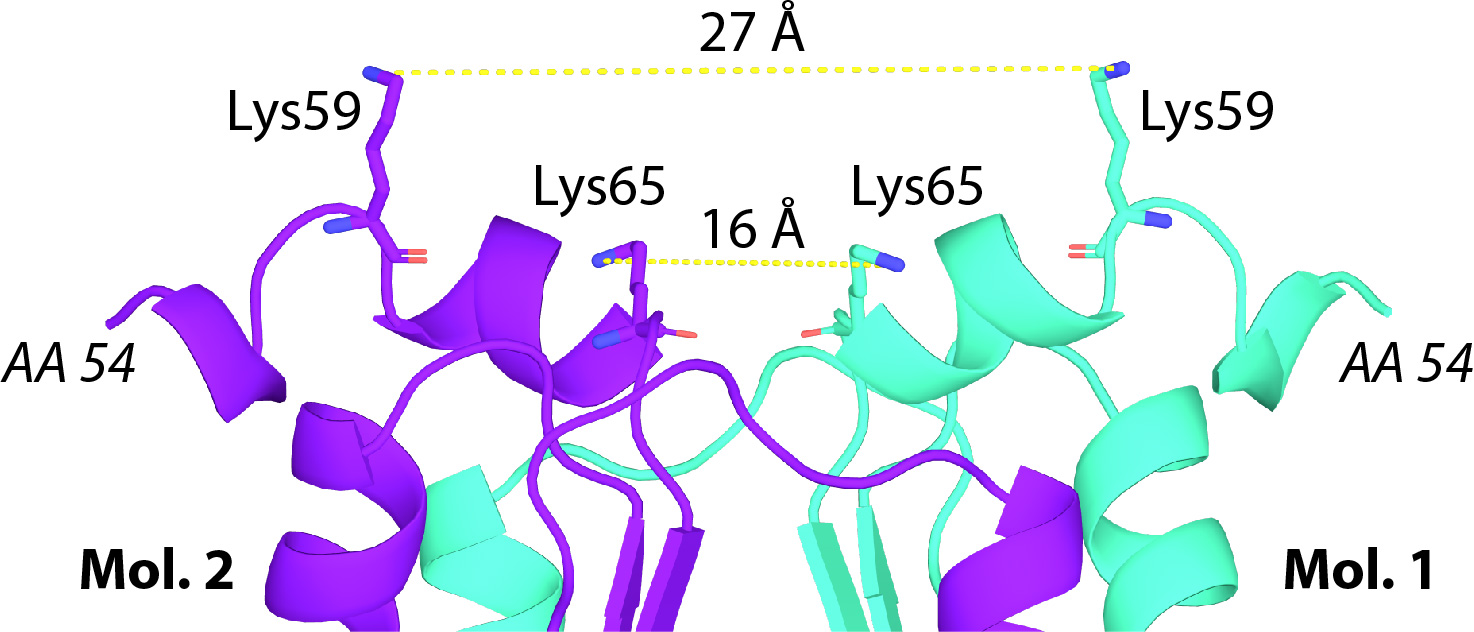

Supplement: FIG S4 [file mbio.01480-21-sf004.jpg]

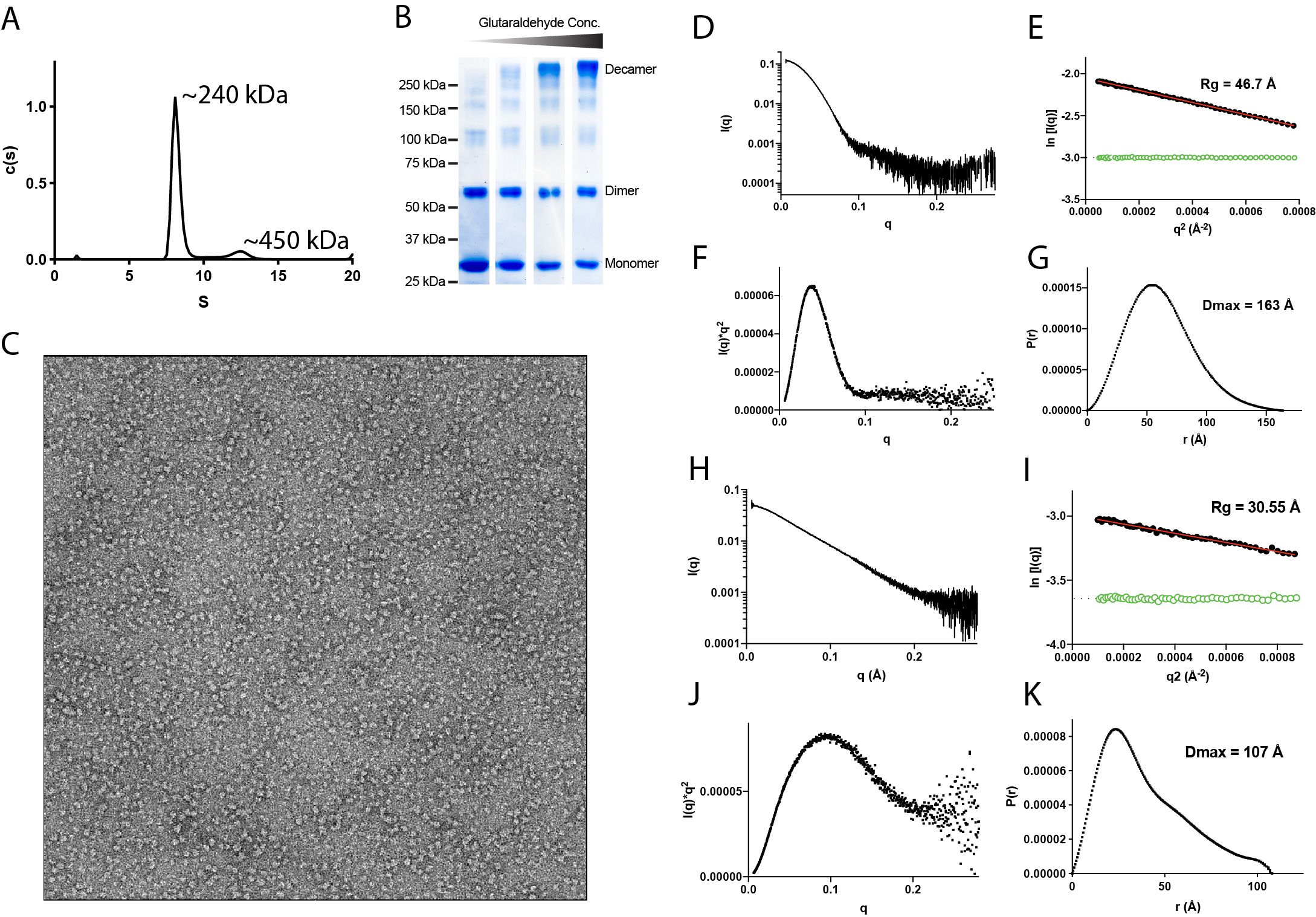

Supplement: FIG S5 [file mbio.01480-21-sf005.jpg]
